# Echocardiographic predictors of severe heart failure symptoms in hypertrophic cardiomyopathy patients with sinus rhythm

**DOI:** 10.1186/1745-6215-9-11

**Published:** 2008-02-29

**Authors:** Fatih Bayrak, Gokhan Kahveci, Muzaffer Degertekin, Bulent Mutlu

**Affiliations:** 1 Yeditepe University Hospital, Department of Cardiology, Istanbul, Turkey; 2 Rize State Hospital, Department of Cardiology, Rize, Turkey; 3 Kosuyolu Heart and Research Hospital, Department of Cardiology, Istanbul, Turkey

## Abstract

**Background:**

Symptoms in hypertrophic cardiomyopathy (HC) appear to be caused by diastolic dysfunction, myocardial ischemia, left ventricle (LV) outflow obstruction, and atrial fibrillation. However, clinical deterioration and severe heart failure symptoms can be observed in patients without any of these factors. Thus, the aim of this study is to determine the echocardiographic predictors of severe heart failure symptoms in patients with HC.

**Methods and results:**

86 HC patients were compared according to symptomatic status. Patients with severe heart failure symptoms were older, preponderantly female, had more often LV outflow obstruction and mitral regurgitation, longer E wave deceleration time (EDt), higher E/Ea ratios and lower LV tissue Doppler (TD) velocities when compared to rest of the patients. LV outflow obstruction (r = 0.43, R^2 ^= 0.19, p < 0.0001), LV lateral mitral annular systolic TD velocity (LMSa) (r = 0.51, R^2 ^= 0.26, p < 0.006) and EDt (r = 0.55, R^2 ^= 0.30, p < 0.027) were found to be the independent predictors for severe heart failure symptoms in forward stepwise regression.

**Conclusion:**

In HCM patients with sinus rhythm and normal LV systolic function, LMSa, EDt and LV outflow obstruction are independent predictors of heart failure symptoms. Diastolic dysfunction determined with EDt, occult systolic dysfunction which is detected with TD analysis, and afterload increase as result of LV outflow obstruction seem to be the main echocardiographic factors affecting symptomatic status in HCM patients with sinus rhythm and normal systolic function.

## Background

Hypertrophic cardiomyopathy (HC) is a complex genetic cardiac disorder with heterogeneous clinical course and expression [1-3]. Exertional dyspnea and disability usually occur in the presence of preserved systolic function and a non-dilated left ventricle (LV) [1,4]. Symptoms appear to be caused in large measure by diastolic dysfunction [5-8], myocardial ischemia [9-15], outflow obstruction associated with mitral regurgitation [16,17] and atrial fibrillation [18]. Of these parameters, which predicts heart failure symptoms best is unclear. Besides, clinical deterioration may be observed even in HC patients without any of these factors [1,2]. Thus, the aim of this study is to determine the echocardiographic predictors of severe heart failure symptoms in patients with HC.

## Methods

### Study population

One hundred and ten consecutive patients who were admitted to our echocardiography laboratory and diagnosed as HC were included in the study. The diagnosis of HC was based on the demonstration of a hypertrophied, non-dilated left ventricle (LV) (wall thickness of at least 15 mm) by 2-dimensional echocardiography in the absence of another cardiac or systemic disease capable of producing a similar degree of hypertrophy [3]. A detailed clinical evaluation was performed for each patient after the echocardiographic examination.

Functional capacity was assessed according to the New York Heart Association (NYHA) classification by one investigator without knowledge of the laboratory results. Heart failure symptoms are defined by exertional dyspnea and functional limitation (with or without chest pain) independent of whether episodes of impaired consciousness (syncope or presyncope) have occurred [19,20]. Ten patients with co-morbid cardiovascular, pulmonary, renal conditions and 14 patients with LV systolic dysfunction (LV ejection fraction < 50%), previous septal myectomy, previous alcohol septal ablation and atrial fibrillation were excluded from the study (remaining 86 patients formed the study population). The study complies with the Declaration of Helsinki, was approved by the local ethical committee and each patient gave written informed consent.

### Echocardiographic analysis

A complete echocardiographic examination was performed with a Vivid Five System (GE, Vingmed Ultrasound, Horten, Norway) in each patient at rest by a single blinded observer. LV hypertrophy was assessed with 2-dimensional echocardiography according to published criteria [3]. The greatest thickness measured at any site in the LV wall was considered to represent LV maximal wall thickness [21]. Peak instantaneous LV outflow gradient was estimated under basal conditions with continuous wave Doppler [17].

2-dimensional measurements included LV end-diastolic and end-systolic diameters, posterior wall thickness, interventricular septal thickness, and LV ejection fraction [22]. Mitral inflow Doppler was measured in standard fashion to determine peak E- and A-wave velocities, deceleration time of the transmitral E wave, and isovolumic relaxation time [23]. Mitral regurgitation was assessed by color Doppler velocity mapping as the ratio of the area of the color jet divided by the maximum LA area [24]. Apical 4-chamber views of color 2-dimensional tissue Doppler (TD) images were acquired during end-expiration at a frame rate of 100 to 140 frames per second to minimize background noise. TD digital data were stored and analyzed offline (EchoPac, GE Vingmed). Sample volumes were placed in the inner half of the myocardium on the basal segments of the left ventricle at the septal, lateral walls and of the right ventricle at the lateral wall adjacent to the atrio-ventricular valve hinge points in the apical 4-chamber view [25]. Systolic (Sa), early diastolic (Ea), and late diastolic (Aa) TD velocities were measured for all segments and subsequently averaged over 3 cardiac cycles in accordance with previous reports [23,25]. Transmitral E/Ea ratios of LV (lateral and septal) were calculated for each patient.

### Statistical analysis

Statistical analysis was performed with SPSS 11.5 (SPSS, Chicago, Illinois, USA) software. Data are expressed as mean ± SD. Relevant relationships were tested by χ^2 ^analysis for proportions and unpaired Student's *t *test for continuous variables. For comparison of continuous echocardiographic variables in different NYHA functional classes, one way analysis of variance (ANOVA) was used. Forward stepwise regression was performed to determine predictors of severe heart failure symptoms. A probability value < 0.05 was required for retention within the final stepwise regression model. Statistical significance was taken as *p *< 0.05.

## Results

### Patient characteristics

The pattern of LV hypertrophy was asymmetric septal in 76 patients, apical in 4 patients, concentric in 5 patients and isolated posterior wall hypertrophy in 1 patient. Forty-three patients (50%) had basal resting left ventricular outflow tract obstruction with a peak gradient > 30 mmHg. Thirty patients had a positive family history of HC (36%) and 18 patients had a history of sudden death in first degree relatives (22%). At admission 32 (37%) of the patients were asymptomatic (NYHA class I), 37 (43%) had mild symptoms (NYHA class II) and 17 (20%) had severe symptoms (NYHA class III-IV).

NYHA class of patients correlated with various echocardiographic and clinical parameters such as age (p = 0.02, R: 0.34), left atrium diameter (p = 0.04, R: 0.22), E wave deceleration time (p = 0.001, R: 0.41), LV outflow obstruction (p = 0.001, R: 0.37), and LV lateral mitral annular systolic tissue Doppler velocity (LMSa) (p = 0.001, R: -0.36).

The comparison of demographic and echocardiographic parameters including TD velocities of HC patients with and without severe heart failure symptoms (NYHA class I-II vs. NYHA class III-IV) is presented in Table 1. Patients with severe heart failure symptoms were older, preponderantly female, had more often LV outflow obstruction and mitral regurgitation, longer isovolumetric relaxation times, higher E/Ea ratios and lower TD velocities of LV when compared to patients without heart failure symptoms. Medical therapies of the patients with and without severe heart failure symptoms were similar.

**Table 1:** Comparison of demographic, clinical and echocardiographic variables of HC patients with and without severe heart failure symptoms (NYHA class I-II vs. NYHA class II-IV)

**Variable**	**Severe symptom + (n = 17)**	**Severe symptom – (n = 69)**	**p**
**Demographics**			
Female gender	10 (59%)	17 (25%)	0.003
Age (years)	54 ± 17	43 ± 16	0.03
Blood pressure, mmHg	116/67	114/67	NS
**Medical therapy**			
Beta-blockers	14 (86%)	57 (84%)	NS
Calcium channel blockers	2 (13%)	10 (15%)	NS
**Echocardiographic findings**			
Left atrium, mm	46 ± 6	44 ± 8	NS
LV end-diastolic diameter, mm	38 ± 8	42 ± 5	NS
LV end-systolic diameter, mm	21 ± 5	24 ± 5	NS
Maximal wall thickness, mm	25 ± 6	24 ± 5	NS
Posterior wall diastolic thickness, mm	16 ± 9	13 ± 5	NS
Mitral E velocity, m/s	0.72 ± 0.2	0.77 ± 0.2	NS
Mitral A velocity, m/s	0.88 ± 0.3	0.80 ± 0.3	NS
E deceleration time, ms	267 ± 61	212 ± 76	0.004
IVRT, ms	113 ± 29	108 ± 30	NS
LV ejection fraction, (%)	75 ± 10	76 ± 8	NS
LV outflow obstruction, n (%)	16 (82%)	29 (42%)	0.0001
**Tissue Doppler velocities**			
E/Ea (septal)	33 ± 24	24 ± 17	0.03
E/Ea (lateral mitral)	33 ± 32	18 ± 16	0.004
Lateral mitral Ea, cm/s	3.2 ± 1.4	4.5 ± 2.5	0.007
Lateral mitral Aa, cm/s	4.0 ± 2.3	5.4 ± 2.9	0.05
Lateral mitral Sa, cm/s	3.1 ± 1.4	4.7 ± 1.9	0.001
Septal mitral Ea, cm/s	2.8 ± 1.5	3.5 ± 1.9	NS
Septal mitral Aa, cm/s	4.7 ± 1.9	5.5 ± 1.9	NS
Septal mitral Sa, cm/s	3.7 ± 1.3	4.5 ± 1.4	0.03
Lateral tricuspid Ea, cm/s	6.7 ± 2.5	7.7 ± 3.0	NS
Lateral tricuspid Aa, cm/s	8.8 ± 3.5	10.0 ± 3.8	NS
Lateral tricuspid Sa, cm/s	9.7 ± 1.9	10.2 ± 3.1	NS

IVRT indicates isovolumetric relaxation time; LV, left ventricle, NS; non significant.

Severe symptom + is used for NYHA class III-IV patients, severe symptom – for NYHA class I-II patients.

### Predictors of severe heart failure symptoms

Table 2 shows the results of univariate and multivariable analyses for prediction of severe heart failure symptoms. LV outflow obstruction (r = 0.43, R^2 ^= 0.19, p < 0.0001), LV lateral mitral annular systolic tissue Doppler velocity (LMSa) (r = 0.51, R^2 ^= 0.26, p < 0.006) and E wave deceleration time (EDt) (r = 0.55, R^2 ^= 0.30, p < 0.027) were found to be the independent predictors for severe heart failure symptoms in forward stepwise regression.

**Table 2:** Univariate and multivariate relations for prediction of heart failure symptoms

	**Univariate**		**Multivariable**		
	**F value**	**p**	**R^2^**	**wald**	**p**

Age	5.79	0.018	...	...	NS
Female gender	7.13	0.009	...	...	NS
LV outflow obstruction	18.5	0.0001	0.19	9.1	0.0001
Mitral regurgitation	15.4	0.0001	...	...	NS
Left atrium diameter	1.3	0.25	...	...	NS
Maximum wall thickness	0.3	0.50	...	...	NS
E wave deceleration time	7.1	0.009	0.30	4.7	0.027
Lateral mitral Ea, cm/s	4.1	0.045	...	...	NS
Lateral mitral Aa, cm/s	3.2	0.07	...	...	NS
Lateral mitral Sa, cm/s	8	0.006	0.26	6.3	0.006
Septal mitral Sa, cm/s	4.1	0.045	...	...	NS
E/Ea (septal)	3.7	0.05	...	...	NS
E/Ea (lateral mitral)	6.4	0.01	...	...	NS

LV indicates left ventricle; NS, non-significant

LMSa correlated with various echocardiographic and clinical parameters such as left atrium diameter (p = 0.004, R: -0.30), maximal wall thickness (p = 0.001, R: -0.36), and NYHA functional class (p = 0.001, R: -0.36).

EDt correlated with age (p = 0.01, R: 0.27), NYHA functional class (p = 0.0001, R: 0.39), isovolumetric relaxation time (p = 0.02, R: 0.26).

**Figure 1:**
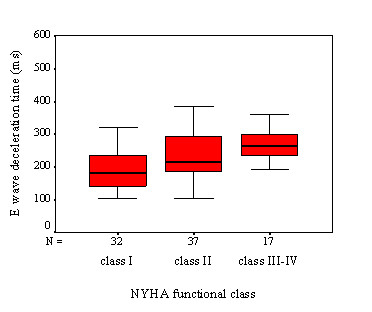
Comparison of E wave deceleration time of severely symptomatic HC patients with the rest of the group (p < 0.001).

**Figure 2:**
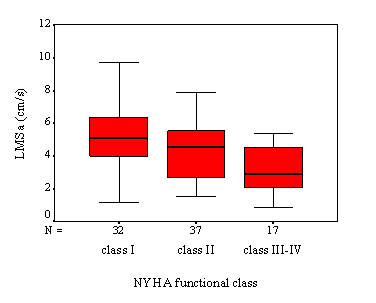
Comparison of left ventricle lateral mitral annular systolic tissue Doppler velocity (LMSa) of severely symptomatic HC patients with the rest of the group (p < 0.002).

EDt gets longer (188 ± 55 ms for class I vs. 233 ± 86 ms for class II vs. 267 ± 61 ms class III-IV, p < 0.001) and LMSa decreases (5.1 ± 2.0 cm/s for class I vs. 4.3 ± 1.7 cm/s for class II vs. 3.1 ± 1.4 cm/s class III-IV, p < 0.002) with increasing NYHA functional class (Figures 1 and 2).

### Effect of LV outflow obstruction on demographic and echocardiographic variables

HC patients with LV outflow obstruction had significantly smaller left ventricle cavities, thicker posterior walls, larger left atrium diameters, longer E wave deceleration times, more often mitral regurgitation and more often severe heart failure symptoms than patients without obstruction. TD velocities of left and right ventricle tended to be lower in HC patients with LV outflow obstruction when compared to patients without obstruction, but that did not reach statistical significance (except lateral mitral Ea).

In 43 patients without LV outflow obstruction, only three patients had severe heart failure symptoms. Severely symptomatic non-obstructive patients had significantly larger left atrium diameters (5.1 ± 0.3 cm vs. 4.1 ± 0.6 cm, p < 0.009) and lower basal septal Ea velocities (1.4 ± 0.8 cm/s for class I vs. 3.8 ± 2 cm/s) when compared to the rest of the non-obstructive HC patients. Because of the low number of severely symptomatic patients in non-obstructive group, no further statistical analyses were performed.

## Discussion

In the present study, we found that NYHA functional class of the HC patients correlated with various echocardiographic and clinical parameters such as age, left atrium diameter, E wave deceleration time (EDt), LV lateral mitral annular systolic tissue Doppler velocity (LMSa), and LV outflow obstruction. Besides these findings, we have shown that EDt, LMSa, and LV outflow obstruction independently predict the presence of severe heart failure symptoms in HC patients with sinus rhythm and normal LV ejection fraction. LMSa is lower and EDt is longer in patients with higher NYHA class which reflects disease severity.

In HC patients, limiting symptoms of exertional dyspnea typically occur in the presence of preserved systolic function and a non-dilated LV [1,4]. Symptoms appear to be caused mainly by diastolic dysfunction [5-8], myocardial ischemia [9-15], outflow obstruction associated with mitral regurgitation [16,17], and atrial fibrillation [18]. On the other side, Chikamori and colleagues suggest that there are different mechanisms of exercise limitation in hypertrophic cardiomyopathy: in patients with a left ventricular outflow gradient at rest, the main determinants of exercise limitation were impaired left ventricular and left atrial systolic performance; in those without a gradient, however, diastolic function at rest was a more important factor in the limitation of exercise performance [26]. In our study, non-obstructive severely symptomatic patients (n = 3) had significantly larger left atrium diameters and lower basal septal Ea velocities when compared with the rest of the non-obstructive patients, but because of the low number of patients, we can not make any further comments. In view of all these information, it still seems to be controversial whether or not left ventricular diastolic pressure and function at rest are major determinants of exercise capacity in hypertrophic cardiomyopathy, and the true mechanism of exercise limitation remains unclear.

Doppler echocardiographic evaluation of trans-mitral LV filling velocities is widely used to assess LV diastolic function. However, most studies [27,28] have failed to show significant correlations between Doppler- derived trans-mitral flow velocities and exercise capacity in patients with HC. Conventional echocardiographic Doppler indexes may be unreliable for assessment of LV diastolic function in such patients, probably because of their dependence on loading conditions [29-31]. In our study, EDt was an independent predictor of severe symptoms but its value may be limited because of its dependence in loading conditions, and as a result tissue Doppler variables and LV outflow obstruction may be more valuable in that respect.

Similar to our findings, the association between tissue Doppler variables and heart failure symptoms were demonstrated in several trials. Tissue Doppler derived indexes were found to be correlated with exercise capacity and NYHA functional class in HC patients [32,33], but these studies have evaluated the relation between symptoms and diastolic tissue Doppler variables. In our study, for the first time we have demonstrated that systolic tissue Doppler variables are also of importance in respect of predicting heart failure symptoms. The decreased LMSa in symptomatic HC patients may be explained with progressive remodelling and/or fibrosis of left ventricle over time and may be of value as a predictor of occult systolic dysfunction and impending heart failure symptoms.

We did not observe statistically significant differences in systolic and diastolic TD velocities of left and right ventricle (except lateral mitral Ea) between HC patients with and without LV outflow obstruction. These findings were in accordance with previously published studies [34,35].

Assessment of symptom severity may be highly subjective, encumbered by the heterogeneous presentation of the patients. So, EDt and LMSa may guide the clinician in deciding the severity of the disease and in making the decision for further invasive therapies. Patients with LV outflow obstruction, longer EDt, and lower LMSa seem to be under risk of clinical deterioration as they have more severe symptoms, but this data will have to be validated with further prognostic studies.

The study was conducted at a tertiary referral cardiac hospital; as a result high prevalence of severe heart failure symptoms (20%) and LV outflow obstruction (50%) were observed.

The small number of HC patients is a weakness of our study, and also we can not exclude the possibility that some patients may develop advanced symptoms of heart failure later in life. NYHA classification, as a subjective measurement of functional disability, may not always reflect functional status accurately in HC patients, but in previous studies, metabolic stress testing in a large HC cohort showed peak oxygen consumption to be significantly related to NYHA class [36].

## Conclusion

For HC patients, LMSa, EDt and LV outflow obstruction are independent predictors of heart failure symptoms. These findings suggest that diastolic dysfunction determined with EDt, occult systolic dysfunction which is detected with tissue Doppler analysis, and afterload increase as result of LV outflow obstruction may be the main echocardiographic factors affecting symptomatic status in HC patients with sinus rhythm and normal systolic function.

## Competing interests

The authors declare that they have no competing interests.

## Authors' contributions

FB carried out the echocardiographic studies, participated in the design of the study and drafted the manuscript. GK carried out the carried out the echocardiographic studies and drafted the manuscript. MD participated in the design of the study and performed the statistical analysis. BM conceived of the study, and participated in its design and coordination and helped to draft the manuscript. All authors read and approved the final manuscript.

## Consent

Written informed consent was obtained from all patients for publication of manuscript. Copies of the written consents are available for review by the Editor-in-Chief of this journal.
